# Phenotyping new rapeseed lines based on multiple traits: Application of GT and GYT biplot analyses

**DOI:** 10.1002/fsn3.3119

**Published:** 2022-11-02

**Authors:** Amir Gholizadeh, Hassan Amiri Oghan, Bahram Alizadeh, Valiollah Rameeh, Kamal Payghamzadeh, Behnam Bakhshi, Seyed Alireza Dalili, Shahriar Kia, Farnaz Shariati

**Affiliations:** ^1^ Crop and Horticultural Science Research Department, Golestan Agricultural and Natural Resources Research and Education Center, Agricultural Research Education and Extension Organization (AREEO) Gorgan Iran; ^2^ Oil Crops Research Department, Seed and Plant Improvement Institute, Agricultural Research Education and Extension Organization (AREEO) Karaj Iran; ^3^ Crop and Horticultural Science Research Department, Mazandaran Agricultural and Natural Resources Research and Education Center, Agricultural Research Education and Extension Organization (AREEO) Sari Iran; ^4^ Crop and Horticultural Science Research Department, Sistan Agricultural and Natural Resources Research and Education Center, Agricultural Research Education and Extension Organization (AREEO) Zabol Iran; ^5^ Plant Protection Research Department, Mazandaran Agricultural and Natural Resources Research and Education Center, Agricultural Research Education and Extension Organization (AREEO) Sari Iran

**Keywords:** GYT biplot, multiple traits, new lines, rapeseed, seed yield

## Abstract

The selection based on multiple traits enhances the crop cultivars merit to farmers. In this regard, 19 breeding lines as well as two commercial cultivars were studied using a randomized complete block design (RCBD) with three replications in three locations during the 2020–2021 growing season. In this study, to identify the association among different traits and to select the best rapeseed lines based on multiple traits, genotype × trait (GT) and genotype × yield × trait (GYT) biplot analyses were used. The results showed that using GYT biplot is more efficient than GT biplot. Based on the GYT biplot and superiority index (SI), the breeding lines G16 and G18 were considered as superior genotypes in combination with the agronomical traits, that is, 1000‐seed weight, number of seeds per pod, number of pods per plant, number of lateral branches, plant height, and pod length with seed yield, which represents a genetic gain in rapeseed breeding program. Based on seed yield combination with phenological traits (early maturity), the breeding line G15 was selected as the best one. Moreover, the line G2 was defined as the superior one in combination of seed yield with pod length. The results indicated that there is a potential for simultaneous genetic improvement of the characteristics (i.e., plant height, number of seeds per pod, early maturity) in rapeseed. Generally, the graphical method of the GYT biplot represented an efficient and practical new way to recognize superior genotypes based on multiple traits in rapeseed breeding programs.

## Graphical abstract

In this study, to identify the association among different traits and to select the best rapeseed lines based on multiple traits, genotype × trait (GT) and genotype × yield × trait (GYT) biplot analyses were used. The results showed that using GYT biplot is more efficient than GT biplot. Generally, the graphical method of the GYT biplot represented an efficient and practical new way to recognize superior genotypes based on multiple traits in rapeseed breeding programs.

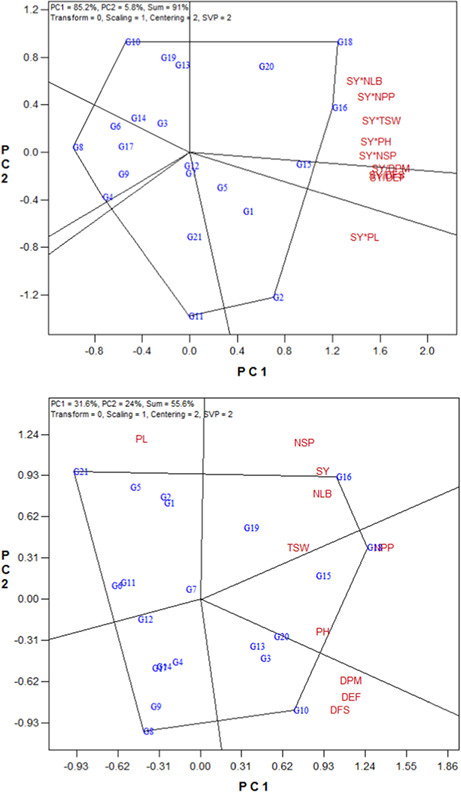

## 1 INTRODUCTION

Rapeseed is the second most important oilseed crop following soybean worldwide that is used in nutritional and pharmaceutical industries (Alizadeh et al., 2021). The seeds of rapeseed encompass oil, vitamin, minerals, and protein as essential materials for nutritional industries (Beyzi et al., 2019). Therefore, the selection of high‐yielding genotypes is especially important in this plant. The selection of high‐yield genotypes is complex and difficult due to low heritability and the existence of the interactions of seed yield (SY) genotype × environment (Gholizadeh & Dehghani, 2016, 2017). Agronomic traits and yield components have relatively high heritability. Therefore, plant breeders prefer selection indirectly using yield components and agronomic traits. The selection based on multiple traits enhances the crop cultivars merit to farmers (Mohammadi, 2019).

There are different methods for examining the relationships between different traits and selecting genotypes based on multiple traits. Among the different methods, the genotype × trait (GT) and genotype × yield × trait (GYT) biplot analyses are advantageous graphical tools that prepare efficient overview of different traits and genotypes relationships. The GT biplot has previously been used in different crops to determine the association between different traits and genotypes such as rapeseed (Dehghani et al., 2008), sorghum (Mukondwa et al., 2021), corn (Santana, Flores, et al., 2021; Santos et al., 2021; Shojaei et al., 2020), white soybean (Yan & Rajcan, 2002), white lupin (Rubio et al., 2004), wheat (Zulfiqar et al., 2021), papaya (Santana, Ramos, et al., 2021), and tomato (Ene et al., 2022).

It is very difficult to develop superior genotypes considering all studied traits. For this purpose, the GYT biplot method recently has been introduced by Yan and Frégeau‐Reid (2018) for the genotypes selection based on multiple traits. The GYT methodology provides a superiority index (SI) for evaluating genotypes based on all yield–trait combinations and identifies the weaknesses and strengths of each genotype (Mohammadi, 2019). This method for the evaluation of the genotypes based on multiple traits was used in different crops by Yan and Frégeau‐Reid (2018) in oats, Kendal (2019) in durum wheat, Boureima and Abdoua (2019) in sesame, Da et al. (2020) in cowpea, Hudzenko et al. (2021) in barley, Peixoto et al. (2022) in cotton, and Gouveia et al. (2020), Gouveia et al. (2022) in *Urochloa* sp. So far, GYT biplot analysis had not been used in rapeseed breeding for examining the relationships between different traits and selecting genotypes based on different traits, and certainly this is the novelty for this paper. Therefore, the aims of this research were (1) to examine the association between different traits and (2) to select superior rapeseed lines based on the combination of agronomic traits with SY.

## 2 MATERIALS AND METHODS

In this study, 19 new rapeseed lines along with Dalgan and RGS003 cultivars were evaluated at three locations with different climates using a randomized complete block design (RCBD) with three replications during growing season (2020–2021). The names and origins of the genotypes are given in Table 1. Table 2 also represents more details of these locations. The plots consisted of four rows of 5 m in length with 30 cm spacing between the rows. The phenological and morphological characteristics and yield components including days to flowering starting (DFS), days to end of flowering (DEF), days to physiological maturity (DPM), plant height (PH), number of lateral branches (NLB), pod length (PL), number of pods per plant (NPP), number of seeds per pod (NSP), 1000‐seed weight (TSW), and SY were measured. Averages of measured traits for three locations in 21 rapeseed genotypes are displayed in Table 3.

**TABLE 1:** Code, name, and pedigree of the tested rapeseed genotypes

Code	Name	Pedigree	Code	Name	Pedigree
G1	SRL‐99‐1	Rameh 97‐10	G12	SRL‐99‐10	Ogh‐Beh‐5 (RGS003* SLM046)
G2	SRL‐99‐2	Ogh‐Beh‐10	G13	SRL‐99‐11	Ogh‐Beh‐6
G3	SRL‐99‐3	Zabol‐6	G14	SRL‐99‐12	Ogh‐Beh‐4
G4	SRL‐99‐4	Ogh‐Beh‐9	G15	SRL‐99‐13	Rameh 97‐1
G5	Dalgan	Dalgan	G16	SRL‐99‐14	Rameh 97‐2
G6	SRL‐99‐5	Ogh‐Beh‐11	G17	SRL‐99‐15	Ogh‐Beh‐3
G7	SRL‐99‐6	Zabol‐9	G18	SRL‐99‐16	Rameh 97‐9
G8	SRL‐99‐7	Ogh‐Beh‐2	G19	SRL‐99‐17	Zabol‐8
G9	SRL‐99‐8	Ogh‐Beh‐7	G20	SRL‐99‐18	Ogh‐Beh‐8
G10	RGS003	RGS003	G21	SRL‐99‐19	LH98‐Rameeh
G11	SRL‐99‐9	Rameh 97‐11 (RGS003* Okapi)			

**TABLE 2:** Agroclimatic characteristics of the locations studied in this research

Location	Longitude (E)	Latitude (N)	Altitude (m)	Minimum temperature (°C)	Maximum temperature (°C)	Average temperature (°C)	Average annual rainfall (mm)	Soil texture	Soil pH	Soil EC (ds/m)	Soil O.C (%)
Sari	53° 10′	36° 41′	29	−5	39	15.1	650	Loamy	7.8	0.64	1.28
Gorgan	54° 20′	36° 55′	5	‐7	42	15.6	460	Silty clay loam	7.3	1.31	1.35
Zabol	61° 32′	31° 50′	489	‐5	41	16.8	50	Sandy loam	8.2	3.00	0.34

**TABLE 3:** Average of measured traits for the three locations in 21 rapeseed genotypes

Genotype	DFS	DEF	DPM	PH (cm)	NLB	PL (cm)	NPP	NSP	TSW (g)	SY (kg.h^−1^)
G1	115	151	182	127	6	7.61	218	24	3.58	2813
G2	115	151	180	137	5	8.11	225	23	3.68	2897
G3	120	160	180	134	5	6.92	230	23	3.92	2538
G4	117	158	183	131	5	7.32	185	23	3.75	2374
G5	114	154	177	123	6	7.42	204	24	3.91	2713
G6	114	154	179	125	6	6.75	186	23	3.81	2375
G7	115	157	181	124	5	6.97	204	23	4.16	2648
G8	117	158	181	132	5	6.76	195	19	3.60	2307
G9	118	156	183	126	5	6.78	200	20	3.50	2492
G10	118	159	186	135	6	6.35	222	22	3.79	2437
G11	116	156	181	130	5	7.78	163	24	3.64	2691
G12	116	156	182	115	6	6.78	191	21	3.61	2725
G13	117	158	182	131	6	6.37	210	22	4.03	2608
G14	116	156	182	126	6	6.74	197	19	3.72	2499
G15	118	159	185	133	6	7.44	230	24	3.79	3007
G16	118	159	183	126	7	7.45	238	28	3.83	3065
G17	114	156	182	130	5	6.62	210	21	3.62	2446
G18	117	159	184	137	7	6.80	239	25	3.88	3099
G19	114	157	181	132	7	7.20	225	25	3.95	2455
G20	117	156	184	130	6	6.36	228	20	3.98	2928
G21	107	154	180	123	6	7.97	195	22	3.84	2600

Abbreviations: DEF, days to end of flowering; DFS, days to flowering starting; DPM, days to physiological maturity; NLB, number of lateral branches; NPP, number of pods per plant; NSP, number of seeds per pod; PH, plant height; PL, pod length; SY, seed yield; TSW, 1000‐seed weight.

### 2.1 Statistical analysis

The data were first analyzed for normality test by Shapiro–Wilk test method using SPSS 19 software (SPSS, 2010). The mean values of the three locations were subjected to statistical analyses for all the traits.

### 2.2 GT biplot methodology

The data were graphically analyzed using the first two principal components derived from singular value decomposition (SVD). The GT biplot was carried out by GGEbiplot software (Yan & Rajcan, 2002). Further information and detailed description on the GT biplot method are available in Yan and Kang′s (2003) review. The results achieved from this analysis were utilized (1) to investigate the interrelationships between different traits and (2) to select superior rapeseed lines based on the multiple traits.

### 2.3 GYT biplot methodology

The GYT biplot methodology was performed according to the procedure of Yan and Frégeau‐Reid (2018). According to this methodology, a trait should be divided or multiplied by SY based on the breeding objectives. The GYT table (Table 4) was achieved by the combination of SY with each trait as follows: for PH, NLB, PL, NPP, NSP, and TSW, the values were multiplied with the yield for each trait (e.g., SY × TSW). While for traits of DFS, DEF, and DPM, in which the low value is desirable, the value for each trait was divided by the yield (e.g., SY/DPM).

**TABLE 4:** Standardized genotype × yield × trait (GYT) values for three locations in 21 rapeseed genotypes

Genotype	SY/DFS	SY/DEF	SY/DPM	SY × PH	SY × NLB	SY × PL	SY × NPP	SY × NSP	SY × TSW	SI
G1	0.72	1.04	0.36	0.42	0.54	1.04	0.60	0.73	0.01	0.61
G2	1.07	1.40	0.86	1.53	−0.31	1.87	1.01	0.64	0.56	0.96
G3	−0.91	−0.73	−0.71	−0.06	−0.94	−0.49	0.28	−0.19	−0.11	−0.43
G4	−1.33	−1.27	−1.60	−0.87	−1.23	−0.57	−1.27	−0.57	−1.08	−1.09
G5	0.39	0.40	0.26	−0.24	0.33	0.53	−0.05	0.49	0.51	0.29
G6	−1.06	−1.01	−1.36	−1.27	−0.39	−1.10	−1.25	−0.57	−0.95	−1.00
G7	0.01	−0.09	−0.29	−0.39	−0.75	−0.14	−0.19	0.06	0.89	−0.10
G8	−1.61	−1.54	−1.77	−1.05	−1.35	−1.28	−1.16	−1.65	−1.64	−1.45
G9	−0.92	−0.66	−1.09	−0.79	−1.03	−0.76	−0.64	−1.05	−1.25	−0.91
G10	−1.15	−1.07	−1.49	−0.37	−0.26	−1.32	−0.18	−0.67	−0.77	−0.81
G11	0.10	0.16	−0.10	0.22	−0.67	0.85	−1.28	0.43	−0.25	−0.06
G12	0.24	0.30	−0.02	−0.81	0.35	−0.13	−0.40	−0.31	−0.21	−0.11
G13	−0.35	−0.32	−0.53	−0.01	0.10	−0.87	−0.11	−0.29	0.42	−0.22
G14	−0.71	−0.63	−1.00	−0.76	−0.13	−0.78	−0.70	−1.28	−0.71	−0.75
G15	1.22	1.23	0.99	1.62	0.95	1.42	1.44	1.19	1.24	1.26
G16	1.46	1.46	1.38	1.23	2.16	1.61	1.84	2.56	1.56	1.70
G17	−0.76	−0.85	−1.23	−0.68	−1.11	−1.04	−0.47	−0.89	−1.13	−0.91
G18	1.71	1.60	1.45	2.31	2.24	0.91	1.96	1.72	1.83	1.75
G19	−0.72	−0.87	−1.13	−0.51	0.65	−0.45	−0.06	0.11	−0.34	−0.37
G20	0.99	1.13	0.72	1.08	0.78	−0.07	1.18	−0.17	1.48	0.79
G21	0.64	−0.07	−0.44	−0.63	0.09	0.77	−0.55	−0.31	−0.07	−0.06

Abbreviations: DEF, days to end of flowering; DFS, days to flowering starting; DPM, days to physiological maturity; NLB, number of lateral branches; NPP, number of pods per plant; NSP, number of seeds per pod; PH, plant height; PL, pod length; SI, Superiority index; SY, seed yield; TSW, 1000‐seed weight.

The GYT biplot method was performed in multilocation trials for multitraits data of genotypes. The GYT biplot was carried out by GGEbiplot software. Yan and Frégeau‐Reid (2018) reviewed further information on the GYT biplot method with detailed description. Based on the standardized GYT data, all yield–trait combinations were also examined for their SI (Yan & Frégeau‐Reid, 2018). The standardized values were calculated according to the method used by Yan and Frégeau‐Reid (2018). The standardization was computed using the following formula:

$$P_{\mathrm{ij}}=\frac{T_{\mathrm{ij}-}{\bar{T}}_{j}}{S_{j}}$$

where *P*_*ij*_ is the standardized value of genotype *i* for yield–trait combination *j* in the standardized table, *T*_*ij*_ is the original value of genotype *i* for yield–trait combination *j*, ${\bar{T}}_{j}$ is the mean across genotypes for yield–trait combination *j*, and *S*_*j*_ is the standard deviation for yield–trait combination *j*.

The results obtained from the GYT biplot methodology was used (1) to investigate the interrelationships between different traits and (2) to select superior rapeseed lines based on the yield–trait combinations.

## 3 RESULTS

### 3.1 Principal component analysis for GT data

Data presented in Table S1 and graphically shown in Figure S1 revealed that the principal component analysis grouped the measured variables into three main components, which generally accounted for 68.5% of the total variation of data. The number of significant principal components was selected based on the eigenvalue higher than 1. According to this criterion, the first three principal components were selected because subsequent eigenvalues were all <1. The first, second, and third principal components are accounted for 31.6%, 24.0%, and 12.9%, respectively, of the variation in data. The first principal component includes DFS, DEF, DPM, and NPP. The second principal component included PL, NSP, and SY. The third principal component includes PH, NLB, and TSW.

### 3.2 The GT biplot for grouping the genotypes

**FIGURE 1:**
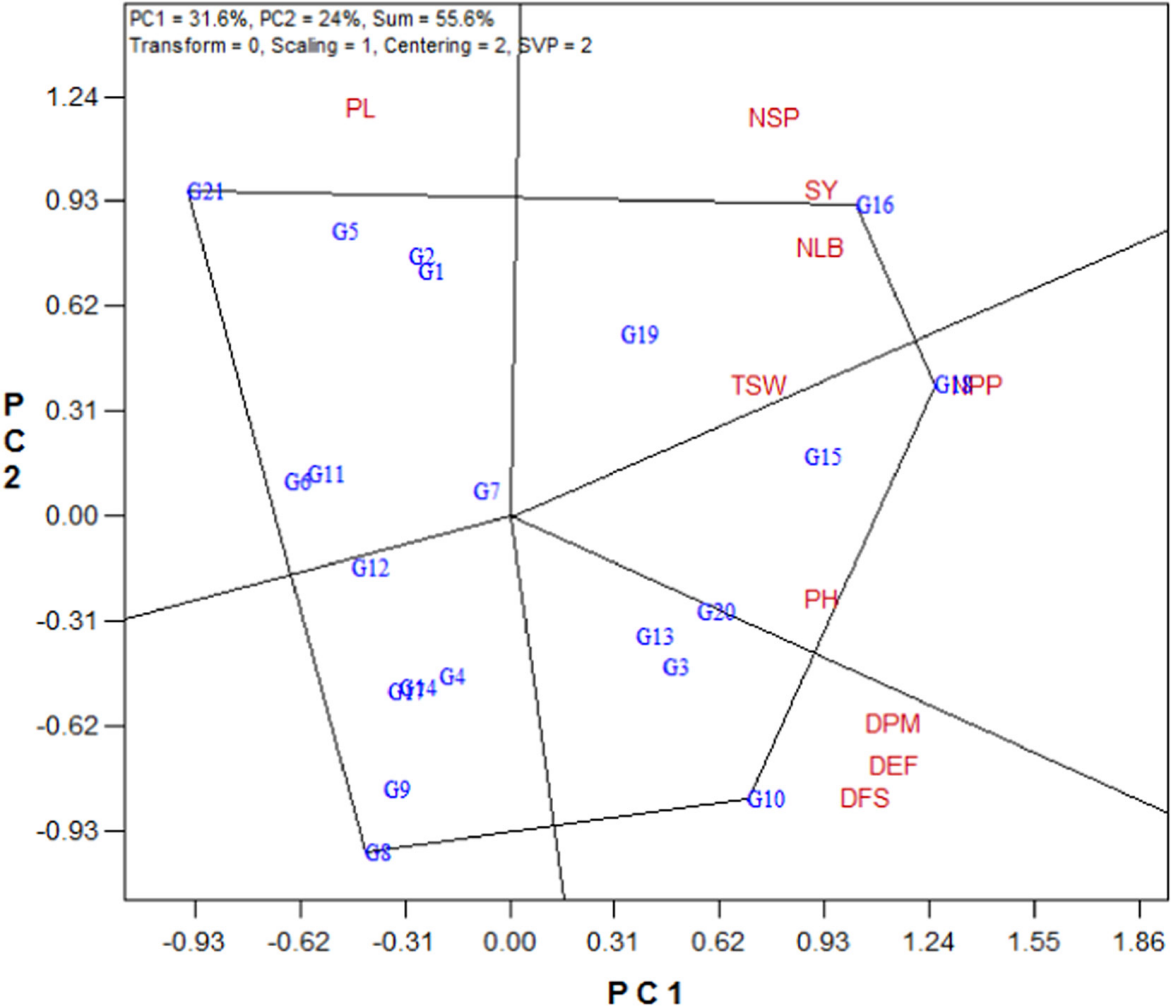
Polygon view of the genotype × trait biplot of rapeseed genotypes. DEF, days to end of flowering; DFS, days to flowering starting; DPM, days to physiological maturity; NLB, number of lateral branches; NPP, number of pods per plant; NSP, number of seeds per pod; PH, plant height; PL, pod length; SY, seed yield; TSW, 1000‐seed weight. Refer to Table 1 for genotypes name.

One of the most useful applications of GT biplot is the polygon view which led to identify the genotypes containing one or more traits with the highest value. The vertex genotype in each sector of the polygon view is the best one in the test trait(s) that falls within that particular sector. According to Figure 1, the vertex genotypes were G21, G16, G18, G10, and G8. The line G16 was the most favorable line for SY, NLB, NSP, and TSW (Figure 1). The line G18 had the highest values for PH and NPP. Also, the cultivar G10 has the highest values for phenological traits (DFS, DEF, and DPM). The vertex line G21 was the favorable line for PL (Figure 1). On the other hand, the vertex line G8 was not favorable in none of the measured traits (Figure 1).

### 3.3 The GT biplot to display the interrelationship among the traits

**FIGURE 2:**
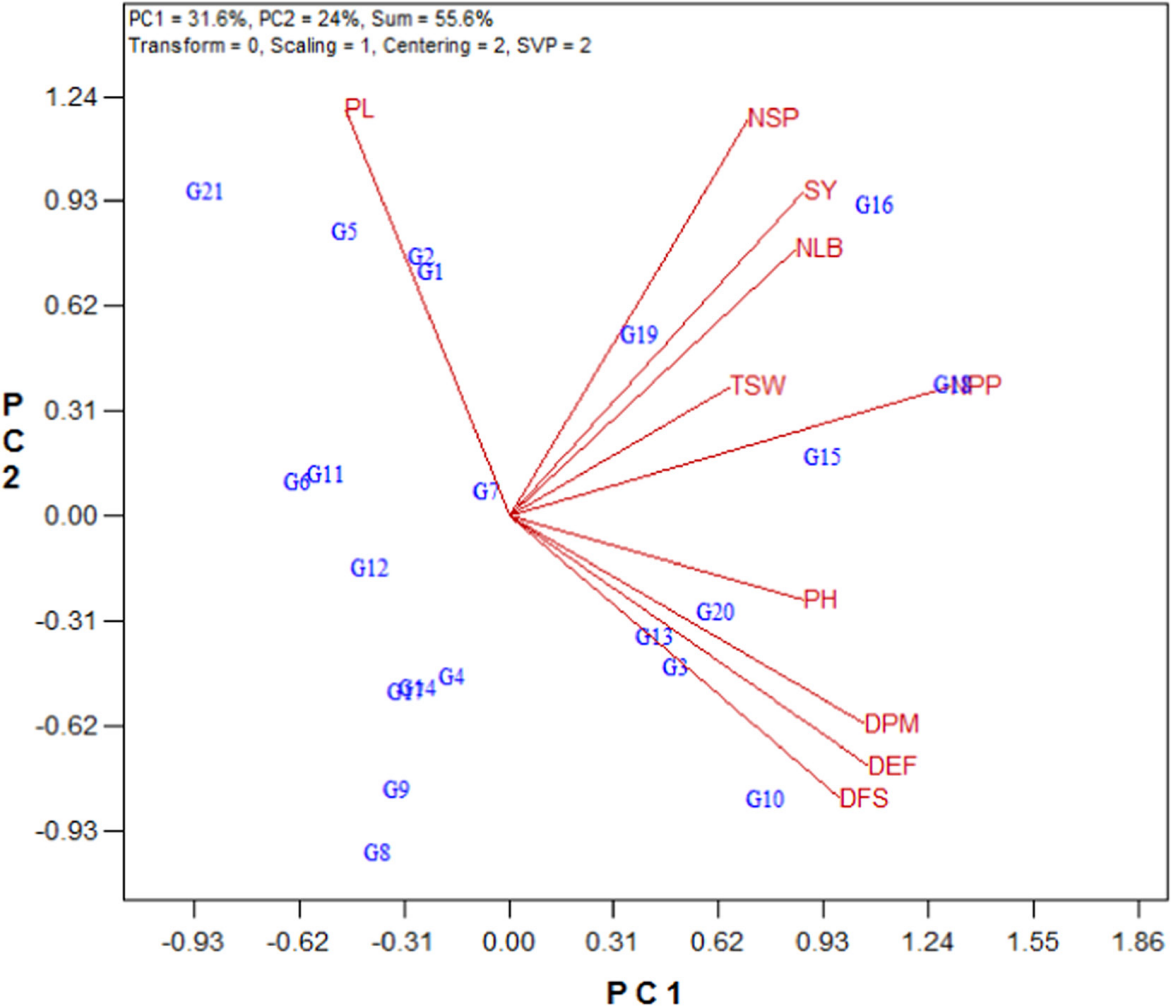
Vector view of the genotype × trait biplot of rapeseed genotypes. DEF, days to end of flowering; DFS, days to flowering starting; DPM, days to physiological maturity; NLB, number of lateral branches; NPP, number of pods per plant; NSP, number of seeds per pod; PH, plant height; PL, pod length; SY, seed yield; TSW, 1000‐seed weight

The vector view of biplot is one of the GT biplot applications to examine the association between environments. In this vector view, each line connect trait to the origin, called the vector. The angle between the vectors of traits shows the correlation coefficients between traits. If the angles of the two traits vectors are <90° and >90°, it indicated a positive and negative correlation, respectively. Also, if the angle is near 90°, it showed that is no relationship. According to Figure 2, there were high positive correlations between DFS, DEF, DPM, and PH and between SY, NPP, NLB, NSP, and TSW.

### 3.4 Principal component analysis for GYT data

Principal component analysis indicated that the first and second principal components were accounted for 91.0% of the total variation in the yield–trait combinations data (Table S2 and Figure S2). The first principal component represents 85.2% of the total variation of the data and this principal component included SY/DFS, SY/DEF, SY/DPM, SY × PH, SY × NSP, and SY × TSW. The second principal component included SY × NLB, SY × PL, and SY × NPP, which accounted for 5.8% of the total variation of the data.

### 3.5 The GYT biplot for grouping the genotypes

**FIGURE 3:**
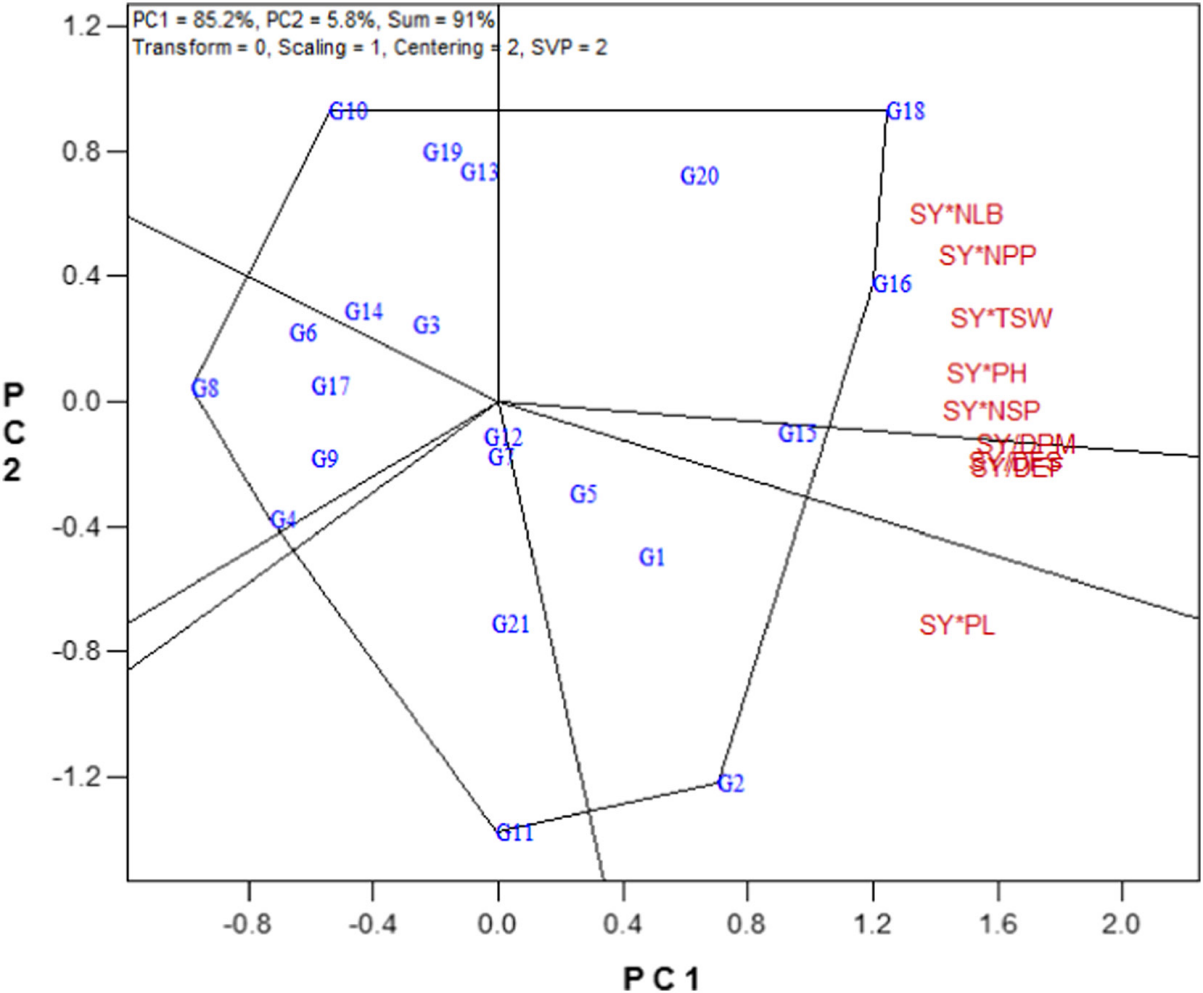
Polygon view of the genotype × yield × trait biplot of rapeseed genotypes. DEF, days to end of flowering; DFS, days to flowering starting; DPM, days to physiological maturity; NLB, number of lateral branches; NPP, number of pods per plant; NSP, number of seeds per pod; PH, plant height; PL, pod length; SY, seed yield; TSW, 1000‐seed weight. Refer to Table 1 for genotypes name.

Figure 3 represents the polygon view of the GYT biplot. According to Figure 3, the breeding lines G16 and G18 had the highest values for SY × NLB, SY × NPP, SY × TSW, SY × PH, and SY × NSP that represent these lines as the best in combining SY with NLB, NPP, TSW, PH, and NSP. The breeding line G15 with the high values of SY/DFS, SY/DEF, and SY/DPM indicated the best combination of SY with phenological traits (DFS, DEF, and DPM). Also, the line G2 with the high value of SY × PL showed the best combination of SY with PL (Figure 3).

### 3.6 The GYT biplot for displaying the relationships among the yield–trait combinations

**FIGURE 4:**
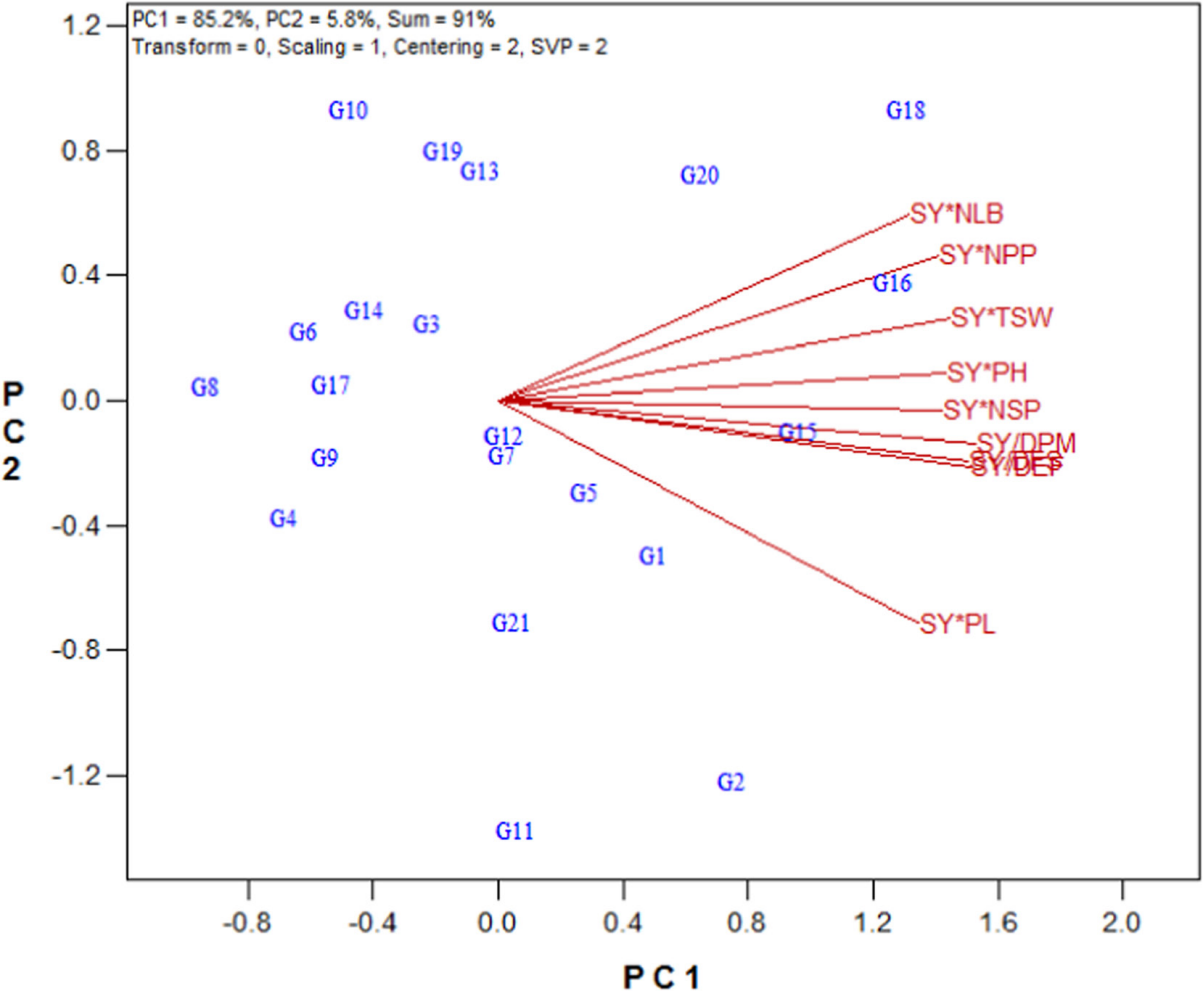
Vector view of the genotype × yield × trait biplot of rapeseed genotypes. DEF, days to end of flowering; DFS, days to flowering starting; DPM, days to physiological maturity; NLB, number of lateral branches; NPP, number of pods per plant; NSP, number of seeds per pod; PH, plant height; PL, pod length; SY, seed yield; TSW, 1000‐seed weight

According to the vector view of the GYT biplot (Figure 4), a positive correlation was observed for all combinations of yield and traits. The most outstanding correlations among traits in the first year were: between SY × NPP and SY × NLB; between SY × PH and SY × NSP; and between SY/DFS, SY/DEF, and SY/DPM, as determined by the acute angles between their vectors (Figure 4).

### 3.7 The GYT biplot view to compare the studied genotypes with the ideal genotype

**FIGURE 5:**
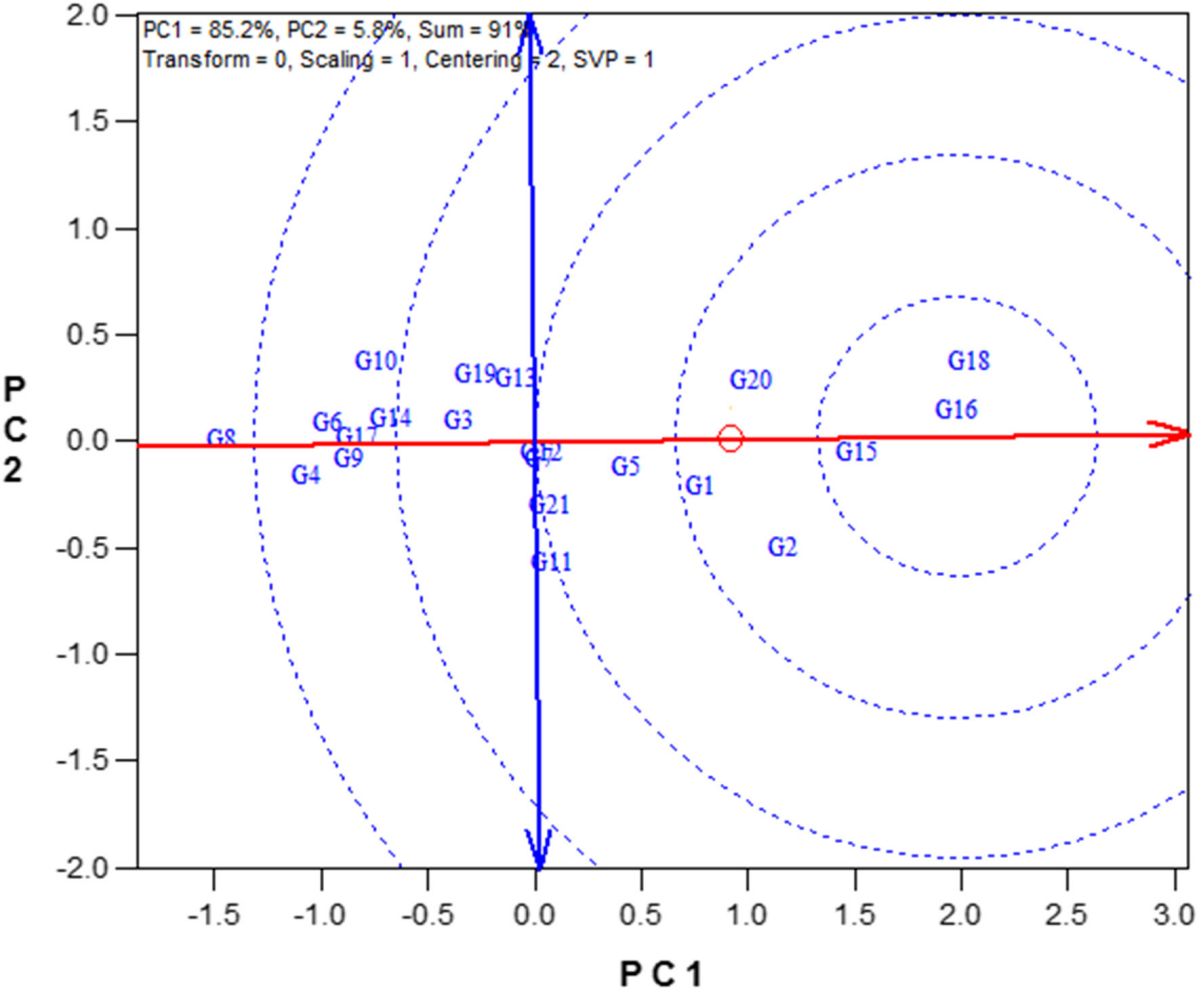
Biplot view of the genotype × yield × trait biplot to compare the studied genotypes with the ideal genotype. Refer to Table 1 for genotypes name.

A ranking biplot that compare genotypes with an ideal genotype is represented in Figure 5. Accordingly, genotypes with the closest distance from the ideal genotype (concentric circles) were considered superior ones. On the other hand, genotypes with the furthest distance to the ideal genotype were nominated as the most undesired ones. Based on Figure 5, the breeding lines G16, G18, and G15 with lowest distance to the hypothetical ideal genotype were defined as the best genotypes, and line G8 due to its maximum distance to the hypothetical ideal genotype is considered as the most unfavorable genotype.

### 3.8 Ranking genotypes based on their superiority

**FIGURE 6:**
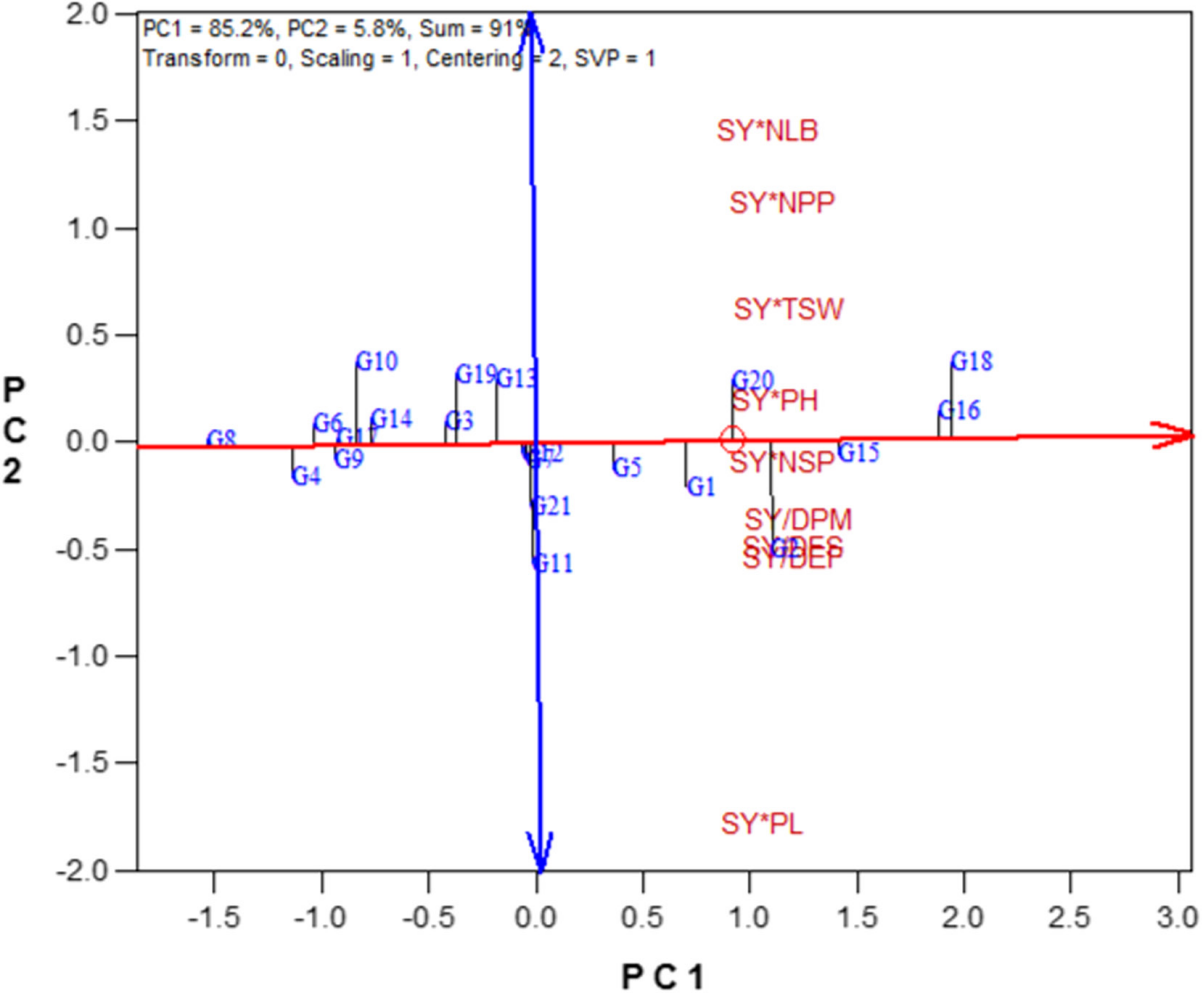
Average tester coordinate (ATC) view of the genotype × yield × trait biplot to rank the genotypes based on the overall superiority and their strengths and weaknesses. DEF, days to end of flowering; DFS, days to flowering starting; DPM, days to physiological maturity; NLB, number of lateral branches; NPP, number of pods per plant; NSP, number of seeds per pod; PH, plant height; PL, pod length; SY, seed yield; TSW, 1000‐seed weight. Refer to Table 1 for genotypes name.

According to the average tester coordinate (ATC) view of the GYT biplot, lines G16 and G18 followed by G15 were defined as the superior ones in terms of all yield–trait combinations, while the weakest line was G8 (Figure 6). Also, based on the SI, the genotypes were ranked considering the combinations of all yield–trait (Table 4). The ranking of genotypes from the most desirable to the most undesirable genotypes is as follows: G18 ˃ G16 ˃ G15 ˃ G2 ˃ G20 ˃ G1 ˃ G5 ˃ G11 ˃ G2 ˃ G1 ˃ G7 ˃ G12 ˃ G13 ˃ G19 ˃ G3 ˃ G14 ˃ G10 ˃ G17 ˃ G9 ˃ G9 ˃ G4 ˃ G8.

## 4 DISCUSSION

Oilseeds breeding and cultivation are necessary to increase yield in Iran because the major percent of vegetable oil is imported to the country. Rapeseed is one of the most important oilseed plants that is widely adapted to the climatic conditions of Iran (Alizadeh et al., 2021). Therefore, it is necessary to develop new high‐yielding genotypes through rapeseed breeding. There are two main issues for developing new cultivars. The first one is the existence of genotype × environment interaction (Ghaffari et al., 2021). To overcome this problem, the researchers evaluated new cultivars in different years or locations. The next issue is the development of new cultivars with high potential in terms of all agronomic traits. The selection of new cultivars is usually based on yield. On the other hand, yield is a quantitative complex trait with low heritability and yield‐based selection may not be effective. Therefore, in selecting the new cultivars, the other agronomic traits should be considered (morphological, agronomical, and physiological traits).

There are numerous statistical methods for simultaneous selection for yield and other agronomic traits. Among the numerous statistical methods, the GT and GYT biplot methods are very effective graphical tools due to efficient overview of the original data. The GT and GYT biplot methods are excellent tools for graphical examination of superior genotypes, traits, and grouping of genotypes and traits compared with conventional statistical techniques such as ANOVA, mean comparisons, linear correlations, and other complex methods like path coefficient analysis (Gholizadeh & Dehghani, 2016). The GT biplot method has previously been used to compare genotypes based on multiple traits and to understand the interrelationship between yield and traits (morphological, physiological, and quality characters) in different crops (Dolatabad et al., 2010; Malik et al., 2014; Santana, Flores, et al., 2021; Santana, Ramos, et al., 2021; Santos et al., 2021; Yan & Rajcan, 2002). A potential constraint of the GT biplot method is that it may fail to explain most of the variation and therefore fail to display all patterns of the data and this is most likely to occur with large datasets, small main effects, and complex interactions. Even when this is the case, it can be ensured that the biplot of PC1 versus PC2 still displays the most important linear patterns of the data and the pattern is estimated by the total variation of the tester‐centered data minus the noise, which is estimated by the total degrees of freedom, multiplied by the error mean square and can be estimated from replicated data (Yan & Kang, 2003).

The GT biplot method is not able to distinguish the effect of all the traits on yield combination, while the GYT biplot method recently improved to eliminate this deficiency. The GYT biplot method has been introduced as an effective and comprehensive method that graphically identifies the strengths and weaknesses of each genotype and provides a SI for the evaluation of genotypes based on combining all the traits with yield (Kendal, 2019; Yan & Frégeau‐Reid, 2018). This methodology was used by a few researchers for evaluating genotypes based on multiple traits combined with SY, and so far had not been used in rapeseed breeding programs, and certainly, this is the novelty for this paper.

The results showed that the use of the GYT biplot method has more privilege than the GT biplot method in rapeseed breeding studies. Based on the GYT biplot and SI, the breeding lines G16 and G18 were ranked as the superiors in the yield combination with agronomical traits, that is, PH, NLB, PL, NPP, NSP, and TSW with SY. The breeding line G15 was the best in combining SY with phenological traits (DFS, DEF, and DPM). Also, the line G2 was the superior one in SY combination with PL. In contrast, G8 was ranked the poorest. Selection of the superior genotypes based on multiple traits could enhance the value of crop cultivars to farmers. Hence, the breeding lines G16, G18, and G15 can be recommended for cultivation. Also, according to the GYT biplot, there was a positive correlation between all yield–trait combinations. This is an advantage of the GYT biplot method in comparison with the GT biplot. The other benefit of the GYT biplot is that it could be utilized in excessive features identification that decrease costs in traits measuring of field experiments without compromising accuracy. Therefore, a high positive relationship between NLB and NPP shows that one (i.e., NLB) of these traits can be used for the selection criterion. Similarly, the high relationship between PH, NSP, DFS, DEF, and DPM indicates that one (i.e., DPM) of these traits is a suitable selection criterion.

## 5 CONCLUSION

This study is unique, in that the GYT biplot method is used as a comprehensive and effective method for the evaluation and selection of rapeseed lines based on multiple traits. The results indicated that genotype selection could be possible considering multiple traits that led to enhance genetic material in rapeseed breeding programs. The breeding lines G16, G18, and G15 ranked as the superior ones in the combination of all agronomical traits with SY, showing a genetic gain in the rapeseed breeding program. Also our results revealed simultaneous potential genetic improvement of the traits (i.e., PH, NSP, early maturity) in rapeseed.

## FUNDING INFORMATION

This research received no specific grant from any funding agency in the public, commercial, or not‐for‐profit sectors.

## CONFLICT OF INTEREST

The authors declare no conflict of interest.

## ETHICS STATEMENT

This study does not involve any human or animal testing.

## INFORMED CONSENT

Written informed consent was obtained from all study participants.

## Supporting information


Appendix S1

## Data Availability

The data that support the findings of this study are available from the corresponding author upon reasonable request.
