# Supplementary material for: Phenotyping new rapeseed lines based on multiple traits: Application of GT and GYT biplot analyses
Source: Food Sci Nutr. 2022 Nov 2;11(2):853–62. doi: 10.1002/fsn3.3119 (PMC9922118; doi:10.1002/fsn3.3119)
Supplement: Supplementary file 1 — Appendix S1 [file FSN3-11-853-s001.doc]

| 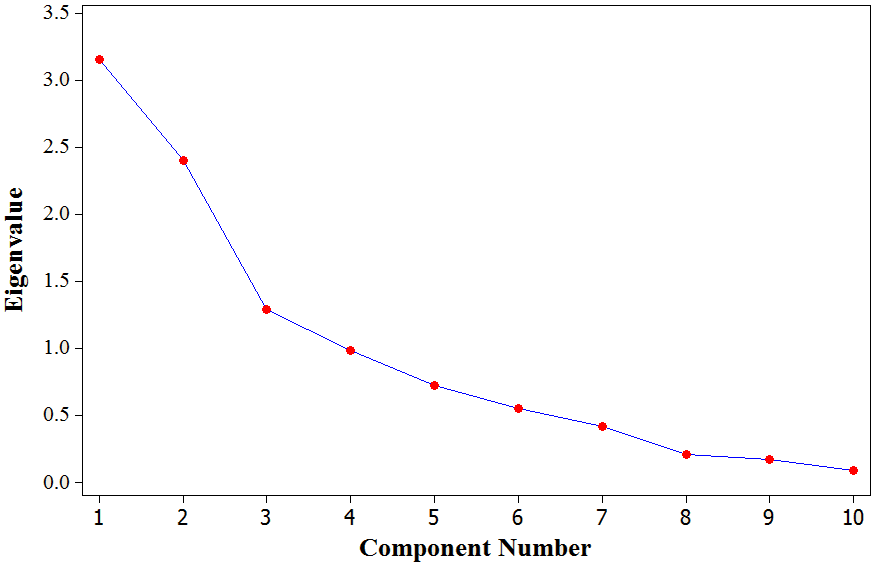 |
| --- |
| **SUPPLEMENTAL Figure 1** Scree plot showing eigenvalues in response to number of components for the estimated variables of rapeseed. |

| 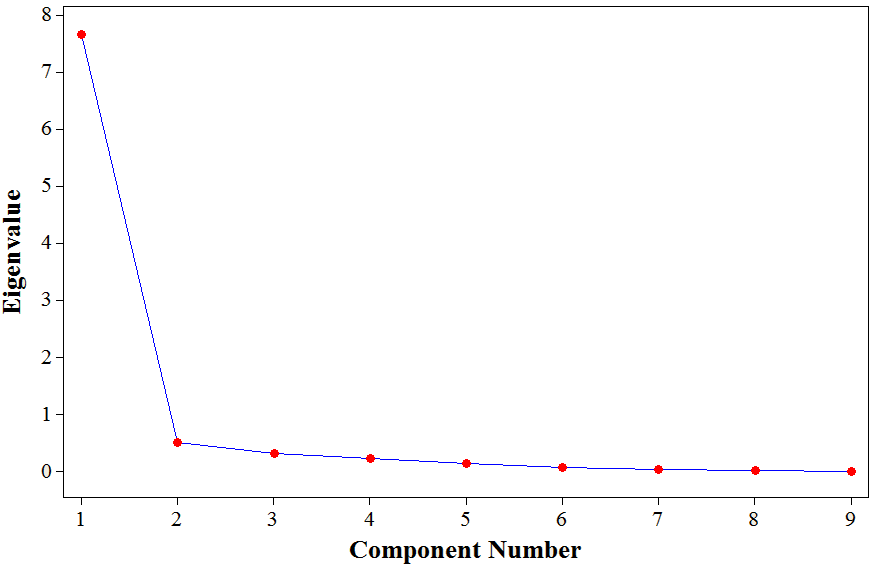 |
| --- |
| **SUPPLEMENTAL Figure 2** Scree plot showing eigenvalues in response to number of components for the estimated yield-trait combinations of rapeseed. |

| **Supplemental Table 1** Eigenvalue of the correlation matrix for the estimated variables of rapeseed using the principal component procedure. | | | |
| --- | --- | --- | --- |
| **Variable** | **PC1** | **PC2** | **PC3** |
| Days to flowering starting | -0.34 | -0.33 | -0.24 |
| Days to end of flowering | -0.37 | -0.30 | 0.20 |
| Days to physiological maturity | -0.37 | -0.25 | -0.21 |
| Plant height | -0.30 | -0.10 | -0.40 |
| Number of lateral branches | -0.29 | 0.31 | 0.34 |
| Pod length | 0.17 | 0.48 | -0.41 |
| Number of pods per plant | -0.45 | 0.15 | -0.06 |
| Number of seeds per pod | -0.25 | 0.46 | -0.05 |
| Thousand seed weight | -0.23 | 0.15 | 0.60 |
| Seed yield | -0.30 | 0.38 | -0.21 |
| Eigenvalue | 3.16 | 2.40 | 1.29 |
| Proportion (%) | 31.60 | 24.00 | 12.90 |
| Cumulative (%) | 31.60 | 55.60 | 68.50 |

| **Supplemental Table 2** Eigenvalue of the correlation matrix for the estimated yield-trait combinations of rapeseed using the principal component procedure. | | |
| --- | --- | --- |
| **Yield-trait combination** | **PC1** | **PC2** |
| SY/DFS | -0.35 | 0.18 |
| SY/DEF | -0.35 | 0.20 |
| SY/DPM | -0.36 | 0.13 |
| SY*PH | -0.33 | -0.07 |
| SY*NLB | -0.31 | -0.53 |
| SY*PL | -0.31 | 0.64 |
| SY*NPP | -0.33 | -0.41 |
| SY*NSP | -0.33 | 0.03 |
| SY*TSW | -0.34 | -0.22 |
| Eigenvalue | 7.67 | 0.52 |
| Proportion (%) | 85.20 | 5.80 |
| Cumulative (%) | 85.20 | 91.00 |
| DFS: Days to flowering starting, DEF: Days to end of flowering, DPM: Days to physiological maturity, PH: Plant height, NLB: Number of lateral branches, PL: Pod length, NPP: Number of pods per plant, NSP: Number of seeds per pod, TSW: Thousand seed weight, SY: Seed yield. | | |
